# Supplementary material for: Mesoscopic insights into effects of electric field on pool boiling for leaky dielectric fluids
Source: Commun Phys. 2025 Apr 30;8(1):188. doi: 10.1038/s42005-025-02102-4 (PMC12043509; doi:10.1038/s42005-025-02102-4)
Supplement: Supplementary file 2 — Supplementary information for [file 42005_2025_2102_MOESM2_ESM.pdf]

Supplementary information for

Mesoscopic insights into effects of electric field on pool boiling for leaky dielectric fluids

Geng Wang[1], Junyu Yang[2], Timan Lei[3], Linlin Fei[4], Xiao Zhao[1], Jianfu Zhao[1,5], Kai Li[1,5*],

Kai H. Luo[3+]

1. National Microgravity Laboratory, Institute of Mechanics, Chinese Academy of Sciences, Beijing 100190, China
2. Institute for Multiscale Thermofluids, School of Engineering, The University of Edinburgh, Edinburgh, UK
3. Department of Mechanical Engineering, University College London, Torrington Place, London WC1E 7JE, UK
4. Key Laboratory of Thermo-Fluid Science and Engineering of Ministry of Education, School of Energy and Power Engineering, Xi'an Jiaotong University, Xi'an, Shaanxi 710049, China
5. School of Engineering Science, University of Chinese Academy of Sciences, Beijing 100049, China

* Corresponding Authors:

Email: likai@imech.ac.cn

+ Corresponding Author:

Email: k.luo@ucl.ac.uk

Supplementary

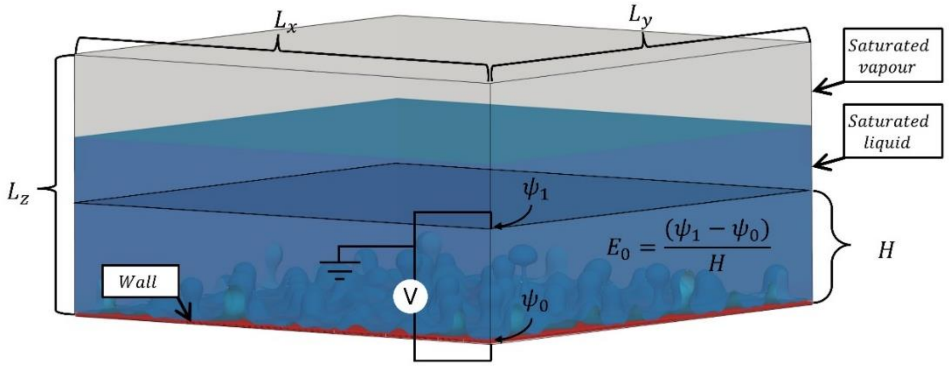

Supplementary Fig. 1. Schematic of the simulation domain

Supplementary Note 1: Model validations.

We start the validation by simulating the evaporation of a stationary droplet in a finite-size system. A droplet with a radius of 35 lattice units is placed at the center of a $200 \times 200 \times 200$ box. We first assess the thermal consistency of our phase-change multiphase model by varying the system temperature from $0.78\,T_c$ to $0.98\,T_c$ and comparing the resulting coexistence densities to those predicted by the Maxwell construction (Supplementary Fig. 2(a)). Additionally, we measure the surface tension at different reduced temperatures $T_r = T_c - T_s$,

It is known that for the van der Waals equation of state, the surface tension $\gamma(\mathrm{T_r})$ follows a scaling law:

$$\gamma(\mathrm{T_r}) \sim (1 - T_r)^{1.5}. \tag{1}$$

Supplementary Fig. 1(b) compares our simulated surface tension values with the theoretical power-law relationship. As shown in Supplementary Fig. 2(a) and 2(b), our simulation results agree well with the theoretical predictions over a wide range of reduced temperatures.

Next, we set the ambient vapor temperature to $\mathrm{T_c}$ and simulate droplet evaporation for three different thermal conductivities λ. The simulation setups are consistent with the test case presented in Fig. 6 of Fei et al.'s work[1]. Supplementary Fig. 2(c) shows the quantitative evolution of the droplet diameter and a comparison with the simulation results from Fei et al.'s work[1]. The corresponding snapshots of the evaporating droplets are presented in Supplementary Fig. 2(d). Our simulation outcomes align closely with those of the previous study, with a maximum difference of less than 5%. This minor discrepancy may be attributed to differences in the initial setup of the temperature field.

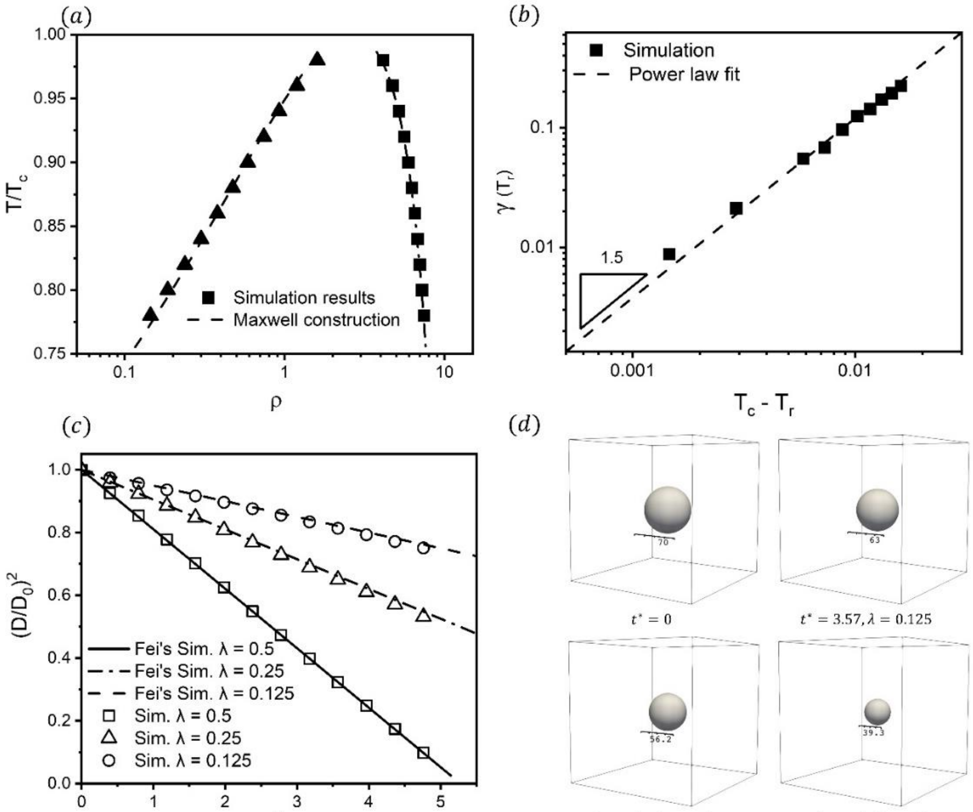

Supplementary Fig. 2. At various T_r, comparison of (a) simulated gas-liquid coexistence density and Maxwell construction, (b) the surface tension with the power law fit equation. (c) The time evolution of droplet evaporation diameter with different thermal conductivities λ, the solid line represents the simulation results from Fei et al.[1], and (d) snapshots of droplet evolution during the evaporation.

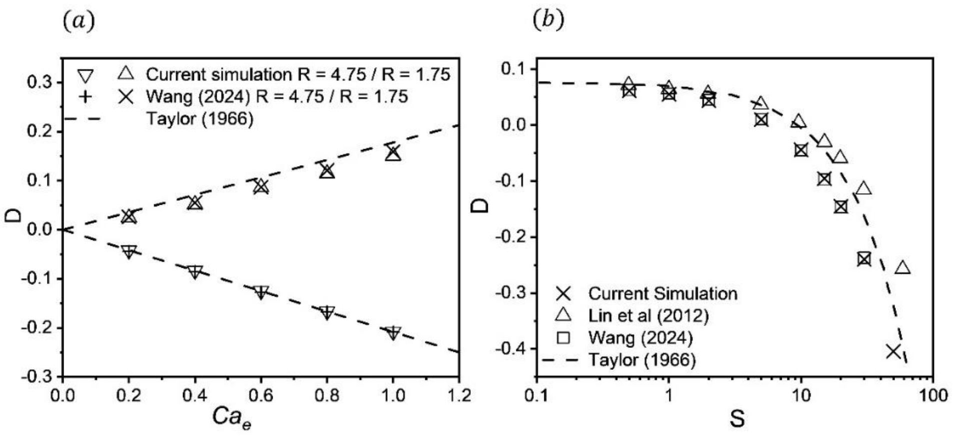

Supplementary Fig. 3. Comparison of Q at steady-state under (a) different R and $\mathrm{Ca_e}$ and (b) different S with theoretical solutions and previous simulations.

We then simulated droplet deformation in an electric field with varying electric capillary numbers ($\mathrm{Ca_e}$), electrical conductivity ratios ($\mathrm{R} = \sigma_l/\sigma_g$), and permittivity ratios ($\mathrm{S} = \varepsilon_l/\varepsilon_g$). The simulation configurations are identical to those used in our recent paper (Case in Fig. 4(a) of our previous work[2]). We compared the droplet deformation factor obtained using the phase-change EHD multiphase model proposed in this study with the original EHD multiphase model from our previous work[2], previous simulation results by Liu et al.[3], and theorical equation[4]. The droplet deformation factor is defined as Q = (L-D)/(L+D), where L and D are the length and diameter of the droplet when it reaches a steady state. As shown in the comparison results in Supplementary Fig. 3(a) and (b), our proposed phase-change EHD multiphase model shows good agreement with previous simulations and theoretical results across a wide range of Ca_e, R and S.

Finally, we reproduced the experiment of bubble shape deformation in an electric field. The simulation setup are listed in Supplementary Table 1, respectively. The bottom wall is set as a hydrophilic surface with a static contact angle of 20°. The qualitative comparisons of stationary bubble shapes between simulation results and experimental results[5] are shown in Supplementary Fig. 4(a), and the quantitative comparison of bubble aspect ratios (Q, the ratio of bubble height to diameter) is presented in Supplementary Fig. 4(b). It can be observed that our simulations align well with the experimental results[5] across a wide range of initial bubble volumes (indicated by Bo) and electrical strength (represented by $\mathrm{Ca_e}$). Some discrepancies are noted for larger bubbles, which may be attributed to differences in surface wettability.

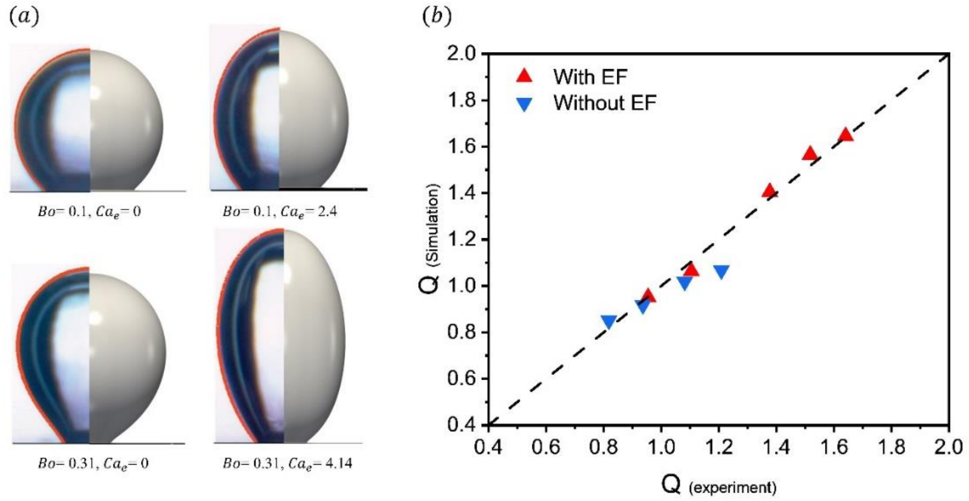

Supplementary Fig. 4. Comparison of simulation results for bubble deformation under different Bo and Ca_e. (a) Qualitative comparison of bubble shape at steady state between current simulation (right white snapshots) and experimental results (left dark snapshots), (b) Comparison of aspect ratios Q between experimental and simulation results.

Supplementary Table 1. Simulation setup of bubble shape deformation in an electric field

Bubble volumes	Bo	Electric field
0.0315	0.059	$Ca_e = 0$
0.0737	0.10	
0.224	0.22	
0.384	0.311	
0.0353	0.063	$Ca_e > 0$
0.0767	0.10	
0.225	0.22	
0.313	0.27	
0.383	0.31	

Supplementary Note 2: Boiling evolution

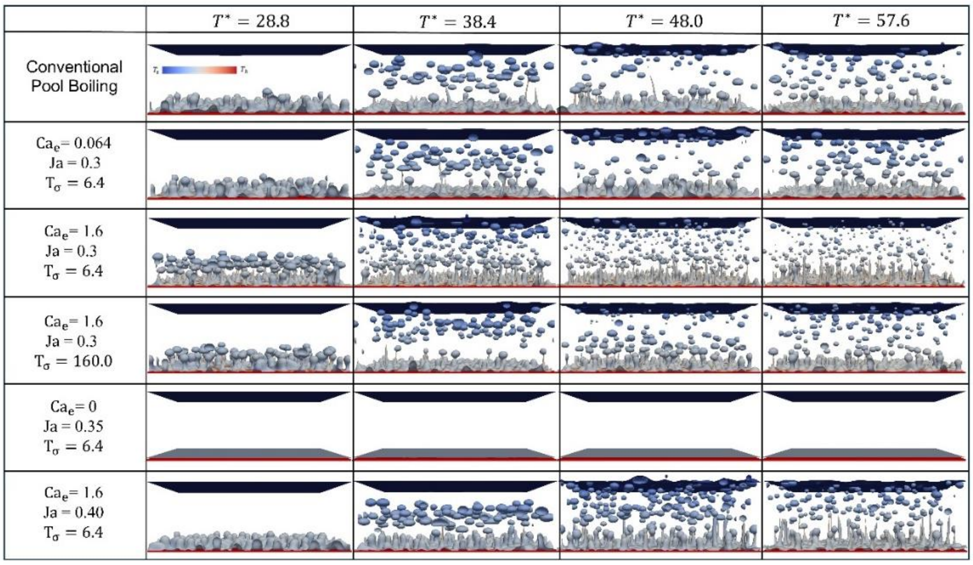

Supplementary Fig. 5. Transient evolution of pool boiling with various operating parameters.

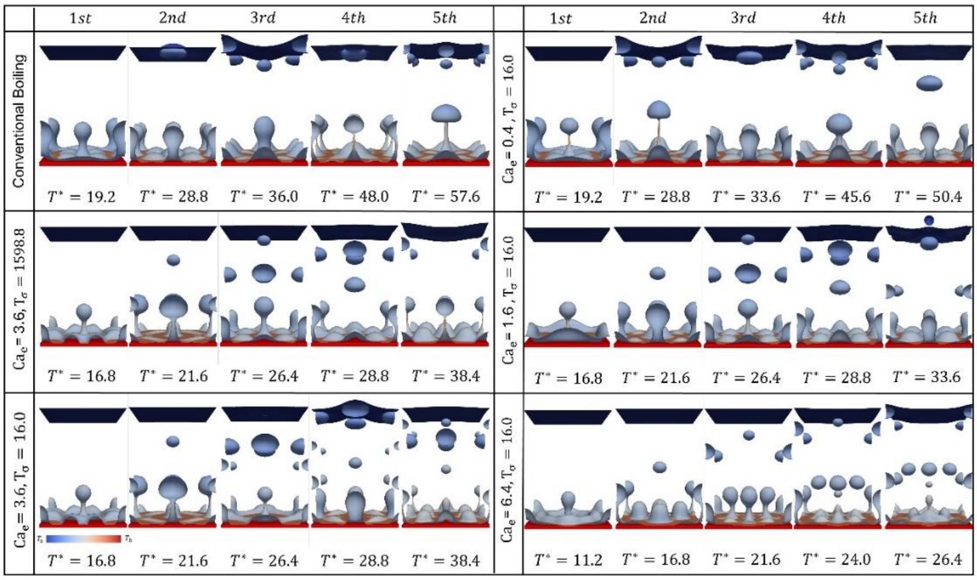

Supplementary Fig. 6. Transient evolution of single bubble boiling under various operating parameters. The contour columns indicate the moments of bubble detachment.

Supplementary References

1. Fei, L., Yang, J., Chen, Y., Mo, H. & Luo, K. H. Mesoscopic simulation of three-dimensional pool boiling based on a phase-change cascaded lattice Boltzmann method. *Phys. Fluids* **32**, 103312 (2020).
2. Wang, G. *et al.* Lattice Boltzmann modelling and study of droplet equatorial streaming in an electric field. *J. Fluid Mech.* **988**, 1–37 (2024).
3. Liu, X., Chai, Z. & Shi, B. A phase-field-based lattice Boltzmann modeling of two-phase electro-hydrodynamic flows. *Phys. Fluids* **31**, (2019).
4. Taylor, G. I., McEwan, A. D. & de Jong, L. N. J. Studies in electrohydrodynamics. I. The circulation produced in a drop by an electric field. *Proc. R. Soc. London. Ser. A. Math. Phys. Sci.* **291**, 159–166 (1966).
5. Di Marco, P., Kurimoto, R., Saccone, G., Hayashi, K. & Tomiyama, A. Bubble shape

under the action of electric forces. *Exp. Therm. Fluid Sci.* **49**, 160–168 (2013).
